# A preliminary assessment of genetic relationships among agronomically important cultivars of black pepper

**DOI:** 10.1186/1471-2156-8-42

**Published:** 2007-06-29

**Authors:** Nisha Joy, Z Abraham, EV Soniya

**Affiliations:** 1 Plant Molecular Biology Lab, Rajiv Gandhi Centre for Biotechnology, Poojappura, Thiruvananthapuram-695014, India; 2 National Bureau of Plant Genetic Resources, Regional Station, Vellanikkara, Trichur-680654, India

## Abstract

**Background:**

The impact of diseases such as Phytophthora foot rot and the replacement of unproductive cultivars by high yielding ones has brought about the disappearance of varieties in *Piper *species, like any other crop. Black pepper (King of spices), is a major spice crop consumed throughout the world. It is widely cultivated across various parts of the world apart from India. The different cultivars may be genetically related and could be a source of valuable genes for disease resistance and an increase in quantity and quality. Even though Western Ghats in India is believed to be the site of origin of this crop, numerous accessions from the NBPGR have not yet been evaluated. Our study aims to investigate the genetic relatedness in major cultivars of black pepper using Amplified Fragment Length Polymorphism.

**Results:**

Amplified Fragment Length Polymorphic (AFLP) DNA analysis was performed in thirty popular cultivars of black pepper from National Bureau of Plant Genetic Resources (NBPGR), India. Fingerprint profiles were generated initially with, five different primer combinations, from which three primer pair combinations (EAGC/MCAA, EAGG/MCTA and EAGC/MCTG) gave consistent and scorable banding patterns. From 173 scorable markers, 158(> 90%) were polymorphic which shows there is considerable variation in the available germplasm. The dendrogram derived by unweighted pair group method analysis (UPGMA) grouped the accessions into three major clusters and four diverse cultivars with only 30% similarity. Karimunda, a widely grown and popular cultivar was unique in the fingerprint profiles obtained.

**Conclusion:**

There are currently few fingerprinting studies using the valuable spice crop black pepper. We found considerable genetic variability among cultivars of black pepper. Fingerprinting analysis with AFLP proved to be an ideal tool for cultivar identification and phylogenetic studies. It shows the high level of polymorphism and the unique characterization of the major cultivars. An extensive range of similarity value between the cultivars was noted (6.01 to 98.13). Further screening of more cultivars will provide valuable information for current breeding programmes.

## Background

The Piper genus includes the most valuable economically important spice crop, black pepper (*Piper nigrum *L) the 'King of Spices' which is consumed throughout the world. Apart from Asian countries such as Malaysia, India, Indonesia, Thailand, Vietnam, China and SriLanka, it is also cultivated in Brazil and Madagascar. The humid climatic conditions and the daily consumption in the diet made this spice crop a synonym of the Asian continent. The Western Ghats of Indian peninsula is the primary centre of origin of the 'King of spices' (*Piper nigrum *L), the source of medicinally and commercially important black pepper [1]. By habit, it is a perennial woody climber. Kerala the southernmost state of India occupies a considerable portion of the Western Ghats and is a rich source of wild relatives of this spice crop. The hot and humid climate of the sub-mountainous tracts of Western Ghats is ideal for its cultivation and hence Kerala is the centre for the production of most of the black pepper in India.

Cytological studies [2-4] suggest that the basic chromosome number of *Piper *is x = 13, whereas *Piper nigrum *is tetraploid (2n = 52). Chance cross-pollination between different species of Piper might have occurred when more than one species climbed up the same support trees. Due to the absence of a pollen transfer mechanism, subsequent gene flow is restricted in these progenies. High successful vegetative propagation ensures further survival and spread of these progenies. The present day *Piper nigrum *cultivars are the descendants of such segregating populations which are vegetatively propagated by farmers through cuttings [1]. Breeding and conservation programmes by humans based on good fruit set, pungency etc, contributes to cultivar diversity.

Amplified Fragments Length Polymorphism (AFLP) is an accepted tool among molecular biologists owing to its high resolution, wide genome coverage and reproducibility. AFLP is a novel PCR based assay [5] for plant DNA fingerprinting that reveals significant levels of DNA polymorphism. Genetic diversity studies by AFLP analysis have been applied to several accessions of plant genera like South Indian tea [6], *Brassica *[7], potato [8], soyabean [9], barley [10], wheat [11], marijuana [12], lupines [13], and opium poppy [14]. Awareness of genetic diversity among the available germplasm is a worthy pre-requisite for crop improvement programmes. To date, little work has been published in fingerprinting studies of black pepper except for Randomly Amplified Polymorphic DNA (RAPD) markers [15]. Even though RAPDs' generate polymorphic markers with random primers, the technique suffers from irreproducibility between laboratories [16,17]. The objective of the present study was to assess the usefulness of AFLP for cultivar identification and to determine genetic relationships of important cultivars of *Piper nigrum*.

## Results

**Figure 2:**
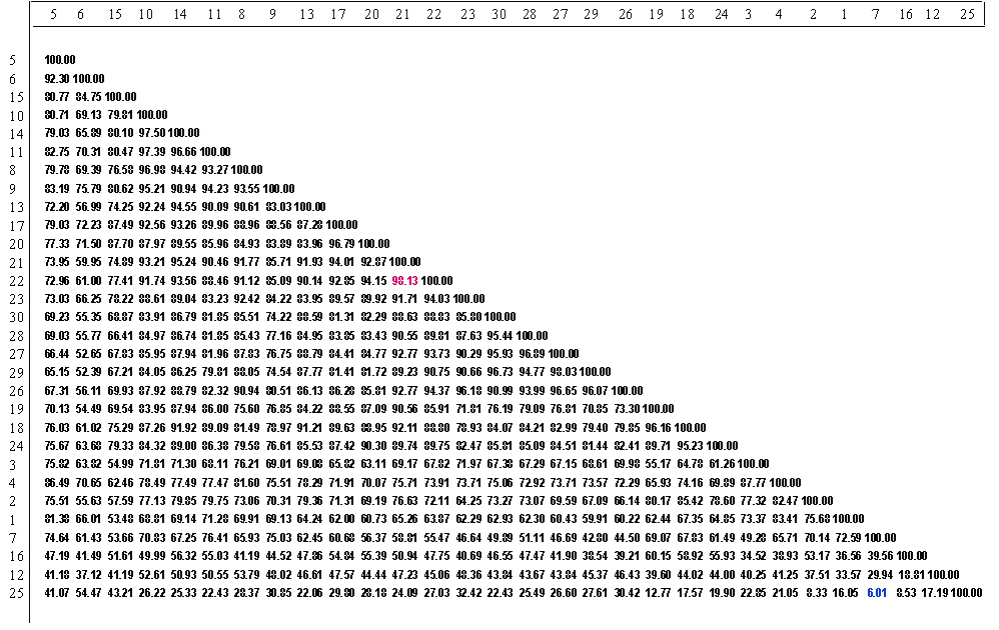
Matrix showing similarity value between the black pepper cultivars (numbers on the left and right border correspond to the serial number as in table 1, indicating the cultivars).

**Figure 3:**
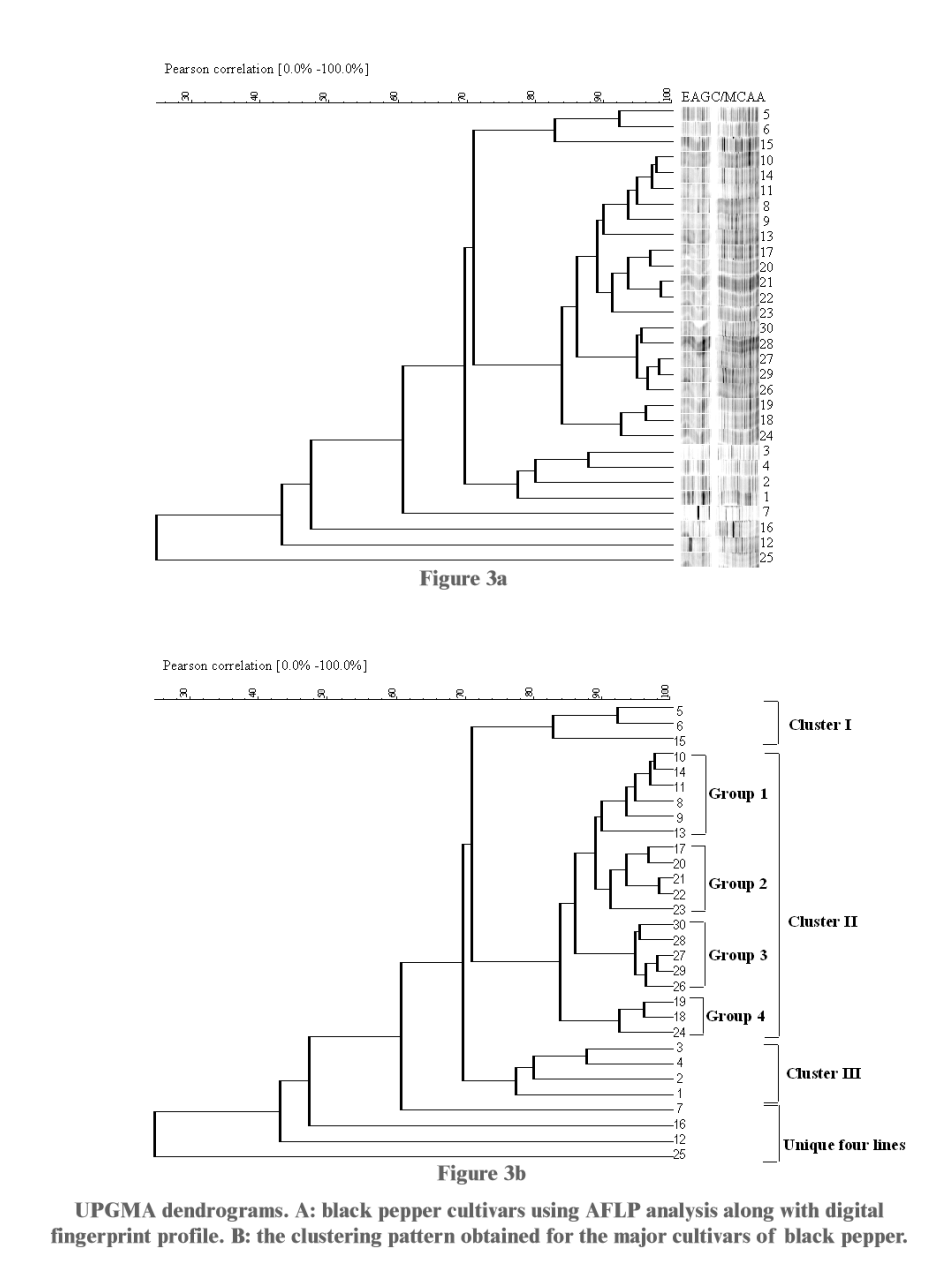
UPGMA dendrograms. A: black pepper cultivars using AFLP analysis along with digital fingerprint profile. B: the clustering pattern obtained for the major cultivars of black pepper.

AFLP analysis generated a large number of reproducible and unambiguous markers for fingerprinting the cultivars of black pepper used in the study. Initially DNA fingerprints were created using five different AFLP primer combinations, from which three were selected for analysis. The three primer pair combinations (EAGC/MCAA, EAGG/MCTA and EAGC/MCTG) resolved 173 markers that could be scored reliably. Across all the thirty cultivars evaluated, 158 markers (91.4%) were polymorphic. The details of the bands scored for further analysis with different primer combinations are illustrated in Table 1. A similarity matrix (Figure 2 and see Additional file 1) and a UPGMA dendrogram (Figure 3a and 3b) were generated from the AFLP analysis software (Bionumerics software package, version 3.0, Applied Maths, Belgium). The dendrogram (Figure 3b) shows three main clusters (I, II and III) and four genetically distinct cultivars. The majority of the cultivars fall into cluster II, which can be further divided into four groups.

**Table 1:** The details of the polymorphic pattern generated by three different primer combinations

Primer pair	Total number of bands	Total number of polymorphic bands	Percentage of polymorphism
EAGC/MCAA	72	59	81.9
EAGG/MCTA	63	62	98.4
EAGC/MCTG	38	37	97.3

Total	173	158	91.4

Our results show that cultivars with the same name from different localities do not always group together. The Vellamundi accessions 14 and 11 are from different localities; both are in cluster II, group1 and have a similarity coefficient of 96.6. Similarly, the Neelamundi accessions 10 and 17 have high similarity (92.56), although they occur in different groups within cluster II. The Thevanmundi accessions 12 (unique) and 15 (cluster I) have a similarity coefficient of 41.19. Likewise, the Karimunda accessions 3 (cluster III) and 16 (unique) have low genetic similarity (34.52). Morphological studies [1] found that the Karimunda cultivar (from Kerala) had distinctive leaf anatomical and stomatal characters. Accession 16 is most dissimilar from the rest and therefore may be a candidate for the "true" Karimunda. Regardless of names, the genetic distinctiveness of accession 16 makes it potentially more interesting for breeding programmes.

Our cluster analysis reveals four distinctive cultivars, accession 7 (local e), 16 (Karimunda), 12 (Thevanmundi) and 25 (Konnamankara). In our results, cultivars from the same locality mostly cluster together – eg. Cluster II, group 3 is made up of cultivars from Panniyur, all of the cultivars from Nedunkandam are in Cluster II, group 1, the Kochandi and Konni cultivars are together in cluster II, group 2, the Puyamkutti cultivars are together in cluster III.

AFLP analysis can provide information about locally derived (unnamed) cultivars. In our results, the local accessions mostly cluster with named accessions, except for local e, which is apparently not closely related to any of the other cultivars. Analysis of further accessions, particularly from the Ayiram Acre locality will help determine whether this cultivar is really genetically unique.

Morphologically the cultivars Kuching (29), Vattamundi (9), Kottanadan (28) and Thevanmundi (12 or 15) have been found to be similar [18,19]. Our AFLP analysis shows that Kuching (29), Vattamundi (9), Kottanadan (28) are all closely related (similarity >70). The Thevanmundi accessions are not as closely related, with Kuching/Thevanmundi 12 having a similarity coefficient of 45.37 and Kuching/Thevanmundi 15 having a coefficient of 67.21. Kuching is the most popular cultivar in Malaysia. It is thought that black pepper was brought to Malaysia from Southern India by the Chinese trader Zheng, who organized a series of expeditions to the Malabar Coast, visiting numerous seacoasts on route [20].

## Discussion

*Piper nigrum *L. has a high level of polymorphism (96.6%) among the cultivars indicating extensive genetic variation of the Indian germplasm [21]. Pradeepkumar et al., 2003 [22] reports the increase in divergence among landraces, compared to the advanced cultivars, that have been derived mostly by clonal selection from land races, though a few are of hybrid origin. Morphologically divergent intraspecific variants of this species occur both in wild forms and in the cultivated varieties. These facts should be considered essentially to select diverse types in crossing programmes for improvement of breeding and while planning conservation strategies.

The AFLP profiles depend mainly on the selective base pairs in the primer, which are utilized to amplify the *EcoR1/MseI *restriction digested DNA fragments. The selective base pairs in the primer correspond to the first base pairs of the genomic DNA beyond the restriction site. Changing these three selective base pairs in the primer can be used as a dynamic tool to scrutinize the genetic diversity within specific plant populations.

Fingerprint profiles generated were best with the three primer combinations EAGC/MCAA, EAGG/MCTA and EAGC/MCTG. In a similar study on Lettuce [23], 320 polymorphic loci were determined using only three AFLP primer combinations. In our study the mean number of bands created was highest (72) for primer pair EAGC/MCAA, when compared to the other two combinations selected (EAGG/MCTA-63, EAGC/MCTG-38). Even though the primer pair EAGC/MCAA had greater number of scorable bands, the number of polymorphic bands was high for EAGG/MCTA-62/63; hence the percentage of polymorphism was maximum (98.74%) for this primer pair selected. A comprehensive analysis was achieved when the individual dendrograms of each primer pair tested were compared with the composite dendrogram of all the chosen three primer pairs. The mean percentages of polymorphism of all the three primer combinations were found to be very high (> 90%). The majority of landraces were distinctly different from advanced cultivars in the molecular profile indicating artificial breeding efforts have brought distinct genetic changes in the species.

During the course of time, human migration from the plains to the hilly tracts of Western Ghats contributed to the spread of certain high yielding cultivars of black pepper. The cultivars were clonally propagated, identified and named by the farmers. These form the basis of the present day landraces in confined areas. Given this history, it is not surprising to find some possible discrepancies in the naming and identification of cultivars. AFLP analysis is an ideal tool for investigating genetic similarity among accessions from the germplasm collection and identifying the most divergent cultivars. AFLP analysis can also provide information on the uniqueness of the unnamed local cultivars.

## Conclusion

Pepper, is a valuable spice crop. AFLP fingerprinting studies circumvents the traditional methods for identification of cultivars, which will take several years. Using incorrectly identified germplasm material for breeding programmes can produce misleading and unsuccessful results. As seen from the preliminary results, AFLP marker has the potential to provide solutions to the presence of duplicates in the collection of germplasm, misnaming of cultivars etc. Thus the data obtained, can also be utilized for further comparison and improvement of pepper cultivars, thereby ensuring a promising future by facilitating rational selection of parents from genetically divergent groups of cultivars. Linkage of genetic markers to specific commercial traits in future will assist plantation management strategies. All the observations support the view that pepper originated in the tropical evergreen forests of Western Ghats and that it is a species that is still in evolution.

## Methods

### Plant material

Thirty accessions of black pepper were obtained from the germplasm collection of National Bureau of Plant Genetic Resource (NBPGR), Thrissur. This represents the most popular widely cultivated landraces. The cultivars extend across the central, southern and northern parts of the state of Kerala (Table 2).

**Table 2:** The descriptive characters of the major black pepper cultivars as provided by NBPGR, Thrissur

**S. No.**	**IC No.**	**Local names**	**Locality**
1	85318	Local a	Kuttampuzha
2	85319	Local b	Karukachal
3	85320	Karimunda	Puyamkutti
4	85321	Local c	Puyamkutti
5	85322	Local d	Patadi
6	85338	Nedumundi	Ayiram Acre
7	85343	Local e	Ayiram Acre
8	85352	Local f	Nedunkandam
9	85354	Vattamundi	Nedunkandam
10	85357	Neelamundi	Nedunkandam
11	85361	Vellamundi	Nedunkandam
12	85371	Thevanmundi	Kumili
13	85375	Narayakkodi	Peermed
14	85376	Vellamundi	Peermed
15	85377	Thevanmundi	Peermed
16	85387	Karimunda	Thadiyanpad
17	85388	Neelamundi	Thadiyanpad
18	85396	Chomala	Mekkazhoor
19	85398	Valiyaramunda	Perinadu
20	85401	Valiyamunda	Kochandi
21	85402	Palikkodi	Kochandi
22	85415	Local g	Konni
23	85420	Karinthakara	Konni
24	85422	Arimani	Adoor
25	85424	Konnamankara	Pandalam
26	85528	Arivally	Panniyur
27	85533	Kanjiramundi	Panniyur
28	85537	Kottanadan	Panniyur
29	85538	Kutching	Panniyur
30	85544	Vokkale	Panniyur

### DNA extraction

From 1 g of young tender leaves of each black pepper cultivar, total genomic DNA was isolated, using Nucleon phtyopure plant DNA extraction kit (Amersham) according to manufacturer's instructions.

### AFLP analysis

DNA fingerprints were generated according to the standard protocol using the AFLP kit (Invitrogen). For each sample, 0.5 *μ*g of DNA was restriction digested with 2.5 *μ*g of *EcoRI/MseI *restriction enzyme in a total reaction volume of 25 *μ*l and incubated at 37°C for 2 hrs. Samples were then transferred to 70°C bath for 15 min before being briefly cooled on ice to inactivate the enzyme. Digested DNA, was supplemented with 25 *μ*l of adapter solution (*EcoRI/MseI *adapters, 0.4 mM ATP, 10 mM Tris-HCl (pH 7.5), 10 mM Mg-acetate, 50 mM K-acetate) and 1 *μ*l of T4 DNA ligase and incubated at 20°C for 2 hrs. This was then diluted (1:10) with TE buffer. 5 *μ*l of diluted ligation mix was combined with 40 *μ*l of preamp primer mix, 5 *μ*l of 10×buffer plus MgCl_2 _and 5 *μ*l of Taq DNA polymerase in order to perform the pre selective amplification. PCR was performed for 20 cycles of 94°C for 30 s, 56°C for 60 s and 72°C for 60 s. Consequently the PCR amplification mixture was diluted (1:50) with TE buffer. For selective amplification, the *EcoRI *primer in each primer pair combination chosen was radiolabelled with [γ 33P] ATP. About 18 *μ*l of *EcoRI *selective primer was labeled with [γ 33P] ATP using 20 *μ*l of T4 polynucleotide kinase (50 mM TrisHCl (pH 7.6), 25 mM KCl, 1 mM β-mercaptoethanol, 0.1 *μ*M ATP, 50% (v/v) glycerol) in 5 × kinase buffer in a total reaction of 50 *μ*l. Labeling was done at 37°C for 1 hr and kept back at 70°C for 10 min for heat inactivation of enzymes. A 50 ul reaction comprising, 5 *μ*l each of diluted pre selective DNA sample, 5 *μ*l of radiolabelled *EcoRI *primer, 5 *μ*l each of *MseI *primer, 20 *μ*l of 10 × PCR buffers with MgCl_2 _and 5 *μ*l of Taq DNA polymerase, was subjected to a touch down PCR of 13 cycles, which included 94°C for 30 s, 65°C for 30 s and 72°C for 60 s. The annealing temperature were reduced by 0.7°C during these 13 cycles in an attempt to give a touchdown phase from 65°C to 56°C. This was followed by 23 cycle reactions of 94°C for 30 s, 56°C for 30 s and 72°C for 60 s. The amplification products were electrophoresed at constant power in a 6% denaturing polyacrylamide gel, to resolve the fragments until xylene cyanol run two-third down the length of the gel. The experiment was repeated to confirm the results. Later the gel was vacuum dried at 80°C for 1 hr and developed using the phosphor imager (Biorad) after exposure for 3–4 hrs.

### Data analysis

Each fingerprint profile was scored for the presence or absence of bands. Very faint bands were excluded and only the scorable, intense bands were used for further analysis. The fingerprint profile with the selected bands alone was further analysed by Bionumerics software package, version 3 (Applied maths, Belgium). A composite dendrogram (for the three primer combinations) and a Pearson correlation coefficient similarity matrix were generated based on the Unweighted Pair Group Method with Arithematic mean (UPGMA).

## Authors' contributions

NJ conducted the experiments and participated in interpretation of results and manuscript preparation. EVS participated in the design of studies and in the discussion for preparing the manuscript and did the final revision. AZ provided the plant materials used for the study. All authors read and approved the final manuscript.

**Figure 1:**
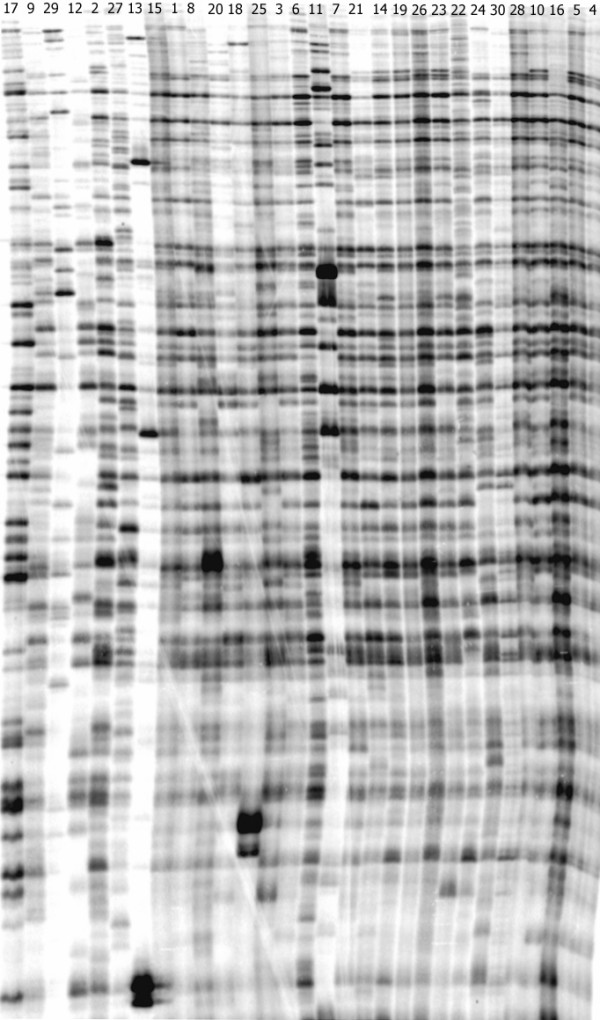
AFLP profile obtained for the black pepper accessions (the number correspond to the serial number as in Table 1, indicative of the cultivars).

## Supplementary Material

Additional file 1 Similarity Matrix. a copy of the distance matrix of Figure 2 in the simple text file showing similarity value between the black pepper cultivars. Click here for file
