# Supplementary material for: A preliminary assessment of genetic relationships among agronomically important cultivars of black pepper
Source: BMC Genet. 2007 Jun 29;8:42. doi: 10.1186/1471-2156-8-42 (PMC1948014; doi:10.1186/1471-2156-8-42)
Supplement: Additional file 1 — Similarity Matrix. a copy of the distance matrix of Figure 2 in the simple text file showing similarity value between the black pepper cultivars. [file 1471-2156-8-42-S1.doc]

**05**  100.00

**06** 92.30 100.00

**15** 80.77 84.75 100.00

**10** 80.71 69.13 79.81 100.00

**14** 79.03 65.89 80.10 97.50 100.00

**11** 82.75 70.31 80.47 97.39 96.66 100.00

**08** 79.78 69.39 76.58 96.98 94.42 93.27 100.00

**09** 83.19 75.79 80.62 95.21 90.94 94.23 93.55 100.00

**13** 72.20 56.99 74.25 92.24 94.55 90.09 90.61 83.03 100.00

**17** 79.03 72.23 87.49 92.56 93.26 89.96 88.96 88.56 87.28 100.00

**20** 77.33 71.50 87.70 87.97 89.55 85.96 84.93 83.89 83.96 96.79 100.00

**21** 73.95 59.95 74.89 93.21 95.24 90.46 91.77 85.71 91.93 94.01 92.87 100.00

**22** 72.96 61.00 77.41 91.74 93.56 88.46 91.12 85.09 90.14 92.85 94.15 98.13 100.00

**23** 73.03 66.25 78.22 88.61 89.04 83.23 92.42 84.22 83.95 89.57 89.92 91.71 94.03 100.00

**30** 69.23 55.35 68.87 83.91 86.79 81.85 85.51 74.22 88.59 81.31 82.29 88.63 88.83 85.80 100.00

**28** 69.03 55.77 66.41 84.97 86.74 81.85 85.43 77.16 84.95 83.85 83.43 90.55 89.81 87.63 95.44 100.00

**27** 66.44 52.65 67.83 85.95 87.94 81.96 87.83 76.75 88.79 84.41 84.77 92.77 93.73 90.29 95.93 96.89 100.00

**29** 65.15 52.39 67.21 84.05 86.25 79.81 88.05 74.54 87.77 81.41 81.72 89.23 90.75 90.66 96.73 94.77 98.03 100.00

**26** 67.31 56.11 69.93 87.92 88.79 82.32 90.94 80.51 86.13 86.28 85.81 92.77 94.37 96.18 90.99 93.99 96.65 96.07 100.00

**19** 70.13 54.49 69.54 83.95 87.94 86.00 75.60 76.85 84.22 88.55 87.09 90.56 85.91 71.81 76.19 79.09 76.81 70.85 73.30 100.00

**18** 76.03 61.02 75.29 87.26 91.92 89.09 81.49 78.97 91.21 89.63 88.95 92.11 88.80 78.93 84.07 84.21 82.99 79.40 79.85 96.16 100.00

**24** 75.67 63.68 79.33 84.32 89.00 86.38 79.58 76.61 85.53 87.42 90.30 89.74 89.75 82.47 85.81 85.09 84.51 81.44 82.41 89.71 95.23 100.00

**03** 75.82 63.82 54.99 71.81 71.30 68.11 76.21 69.01 69.08 65.82 63.11 69.17 67.82 71.97 67.38 67.29 67.15 68.61 69.98 55.17 64.78 61.26 100.00

**04** 86.49 70.65 62.46 78.49 77.49 77.47 81.60 75.51 78.29 71.91 70.07 75.71 73.91 73.71 75.06 72.92 73.71 73.57 72.29 65.93 74.16 69.89 87.77 100.00

**02** 75.51 55.63 57.59 77.13 79.85 79.75 73.06 70.31 79.36 71.31 69.19 76.63 72.11 64.25 73.27 73.07 69.59 67.09 66.14 80.17 85.42 78.60 77.32 82.47 100.00

**01** 81.38 66.01 53.48 68.81 69.14 71.28 69.91 69.13 64.24 62.00 60.73 65.26 63.87 62.29 62.93 62.30 60.43 59.91 60.22 62.44 67.35 64.85 73.37 83.41 75.68 100.00

**07** 74.64 61.43 53.66 70.83 67.25 76.41 65.93 75.03 62.45 60.68 56.37 58.81 55.47 46.64 49.89 51.11 46.69 42.80 44.50 69.07 67.83 61.49 49.28 65.71 70.14 72.59 100.00

**16** 47.19 41.49 51.61 49.99 56.32 55.03 41.19 44.52 47.86 54.84 55.39 50.94 47.75 40.69 46.55 47.47 41.90 38.54 39.21 60.15 58.92 55.93 34.52 38.93 53.17 36.56 39.56 100.00

**12** 41.18 37.12 41.19 52.61 50.93 50.55 53.79 48.02 46.61 47.57 44.44 47.23 45.06 48.36 43.84 43.67 43.84 45.37 46.43 39.60 44.02 44.00 40.25 41.25 37.51 33.57 29.94 18.81 100.00

**25** 41.07 54.47 43.21 26.22 25.33 22.43 28.37 30.85 22.06 29.80 28.18 24.09 27.03 32.42 22.43 25.49 26.60 27.61 30.42 12.77 17.57 19.90 22.85 21.05 8.33 16.05 6.01 8.53 17.19 100.00
